# Graphitization Optimization of Cobalt-Doped Porous Carbon Derived from Seaweed Sludge for Enhanced Microwave Absorption

**DOI:** 10.3390/polym17111572

**Published:** 2025-06-05

**Authors:** Kai Liu, Yusen Ai, Mei Cui, Renliang Huang, Rongxin Su

**Affiliations:** 1 Tianjin Key Laboratory for Marine Environmental Research and Service, School of Marine Science and Technology, Tianjin University, Tianjin 300072, China; 2 State Key Laboratory of Chemical Engineering, Tianjin Key Laboratory of Membrane Science and Desalination Technology, School of Chemical Engineering and Technology, Tianjin University, Tianjin 300072, Chinameicui@tju.edu.cn (M.C.); 3 Zhejiang Institute of Tianjin University, Ningbo 315201, China

**Keywords:** microwave absorption, biomass-derived carbon, cobalt-doped porous carbon, seaweed sludge, graphitization

## Abstract

Utilizing biomass resources to develop carbon-based microwave-absorbing materials adheres to the principles of sustainable development. Nevertheless, the single loss mechanism of pure carbon materials is limited. Additionally, the carbonization of artificially synthesized polymers has poor environmental performance and involves complex processes. These issues restrict their performance and broader applicability. In this study, cobalt-doped seaweed sludge porous carbon (Co/SSPC) with different cobalt contents was synthesized via a simple grinding–carbonization treatment. The addition of cobalt can regulate the graphitization degree of porous carbon, achieving a suitable amorphous-to-crystalline carbon ratio of 2.05. This not only enhances magnetic loss but also modifies dielectric loss and optimizes impedance matching. The construction of synergistic magnetic and dielectric loss mechanisms enables Co/SSPC to exhibit excellent microwave absorption performance. Specifically, Co/SSPC achieved a minimum reflection loss (RL_min_) of −66.91 dB at a thickness of 4.79 mm and an effective absorption bandwidth (EAB) of 5.09 GHz at a thickness of 1.6 mm. This study provides a practical approach for the functional application of natural polymer waste algal sludge and highlights its potential in the low-cost production of microwave absorbing materials.

## 1. Introduction

The extensive use of electronic devices and the continuous progress of communication technologies has raised significant concerns about electromagnetic wave (EMW) pollution, spanning both military and civilian fields [1,2]. This form of pollution poses potential threats to military security, human health, the normal operation of sensitive electronic equipment, and the energy efficiency of communication systems [2,3]. In response to this issue, numerous efforts have been dedicated to developing advanced materials capable of effectively absorbing EMW radiation, which reduce the reflection power by converting the energy of incident EMW into other forms [4,5]. In recent years, EMW-absorbing materials have attracted widespread attention from both academic and industrial fields due to their significant potential in various applications.

Carbon-based materials have emerged as promising candidates for EMW absorption, attributed to their excellent electrical conductivity, a lightweight structure, tunable dielectric constants, and high chemical stability [6,7,8]. Specifically, carbon materials derived from biomass have become particularly appealing due to their sustainability, cost-effectiveness, and abundant availability. Biomass-derived carbon materials are not only environmentally friendly but also contribute to waste reduction, aligning with global initiatives to promote sustainable development [9,10,11,12]. Specifically, carbon materials derived from biomass have become particularly appealing due to their sustainability, cost-effectiveness, and abundant availability, and have demonstrated excellent properties in the fields of pollutant degradation, adsorption, energy, catalysis and so on [13,14,15]. The inherent characteristics of biomass endow a diverse range of microstructures, such as porous architectures and hierarchical frameworks, which are highly advantageous for enhancing electromagnetic absorption capabilities. These materials also offer tunable dielectric properties through controlled processing, rendering them versatile and effective in various applications [16,17,18]. For instance, Zhang et al. [19] reported a highly porous carbon material derived from pumpkin seed shells. The material exhibited good electromagnetic wave absorption performance, highlighting the potential of biomass-derived carbon materials in this field. Lou et al. [20] discussed the application of biomass-based carbon materials in electromagnetic wave absorption, emphasizing their low cost, simple preparation methods, and high effectiveness in both electromagnetic wave shielding and absorption applications. Zhou et al. [21] developed the hierarchical porous carbon materials from waste biomass, showing their potential for electromagnetic wave absorption due to their unique microstructures and tunable properties. Among various biomass resources, marine biomass, especially algal sludges, represents a valuable precursor for synthesizing carbon-based materials. Algal sludges are by-products of the algae industry, with the annual global seaweed waste ranging from 3 million to 5 million tons [22,23,24]. These residues are rich in organic components and inherent minerals, making them ideal candidates for conversion into carbon-based microwave-absorbing materials. During the carbonization process, the minerals present in algal sludges may act as natural dopants, enhancing interfacial polarization. The functional utilization of algal sludges also addresses waste management challenges, creating value-added products while reducing environmental burdens.

As is known, the restricted single-loss mechanism of pure carbon materials limits their EMW absorption performance. The incorporation of transition metals into carbon-based materials has been extensively studied with the aim of enhancing their performance. Transition metals, such as Co, Ni, and Fe, possess unique magnetic properties that complement the dielectric loss mechanisms of carbon materials [25,26,27,28]. In particular, Co has received significant attention due to its ability to introduce magnetic loss mechanisms, such as natural resonance and eddy current loss, as well as enhance interfacial polarization. The synergistic effects of dielectric and magnetic losses can significantly improve the EMW absorption performance of the Co-doped carbon material. For example, Cheng et al. [29] employed hydrothermal synthesis followed by high-temperature carbonization and sulfidation processes to prepare CoFe Metal-Organic Framework (MOF)-derived Co_9_S_8_/FeCoS_2_/C composite materials. This material exhibited excellent EMW absorption performance with a minimum reflection loss (RL_min_) of −53.9 dB. Tian et al. [30] reported a graphite carbon nitride nanotubes/Cobalt@Carbon (GCNNs/Co@C) composite with a RL_min_ of −63.9 dB and a maximum effective absorption bandwidth (EAB_max_) of 4.44 GHz at a thickness of 1.51 mm. Bateer et al. [31] synthesized a composite material of N-graphene and Co nanoparticles (Co/NGN) with excellent dielectric properties through an in situ growth method. This Co/NGN-2 composite achieved an RL_min_ of −45.8 dB and an EAB of 4.6 GHz in the Ku-band (12.4–18 GHz). In addition, Yao et al. [32] synthesized La_2_O_3_/Co@N-doped carbon nanotubes (La_2_O_3_/Co@NCNTs) composites by thermally annealing La [Co(CN)_6_]·5H_2_O Prussian blue analog precursors under an Ar atmosphere. The as-prepared composite exhibited a minimum reflection loss of −53.4 dB and an EAB of 4.34 GHz at a thickness of 2.45 mm. These studies indicate that the incorporation of magnetic metals into carbon-based materials mainly enhances their magnetic losses, and also increases the dielectric losses through the coupling of various mechanisms (such as interfacial polarization), thus effectively improving the ability to absorb electromagnetic waves. However, the exact enhancement mechanism involved in incorporating Co into carbon-based absorbers still remains unclear. In addition, the carbonization of artificial polymers and materials to prepare microwave-absorbing materials has poor environmental sustainability. The preparation process of these microwave-absorbing materials is relatively complex, and the production cost is high. How to achieve low-cost and sustainable large-scale production remains a problem that needs to be solved at present.

In this study, we aim to develop porous carbon composites derived from algal sludges, incorporating cobalt as a dopant to enhance microwave absorption performance, and synthesize the Co-doped seaweed sludge porous carbon (Co/SSPC) by grinding-carbonization process. The microstructures of seaweed sludge porous carbon, combined with the incorporating of Co, are expected to regulate the graphitization degree of porous carbon, as well as generate synergistic effects that improve both dielectric and magnetic losses. A systematic investigation is carried out to analyze the performance of Co/SSPC, focusing on their dielectric and magnetic loss mechanisms. The study explores the underlying absorption mechanisms and evaluates the potential of these materials for practical applications.

## 2. Materials and Methods

### 2.1. Materials

Cobalt nitrate hexahydrate (Co(NO_3_)_2_·6H_2_O, 99%, AR), ethanol(C_2_H_6_O, 95%), and paraffin wax (99%) were purchased from Ningbo Changyuan Instrument Co., Ltd. (Ningbo, China) without further purification. Algal sludge (dark brown powder), a by-product of the seaweed processing, was purchased from Qingdao Huifulin Marine Biotechnology Co., Ltd. (Qingdao, China). Throughout the experiments, deionized water with a conductivity of less than 5 μS/cm was used.

### 2.2. Synthesis of Co-Doped Seaweed Sludge Porous Carbon (Co/SSPC)

The algal sludge was sequentially washed with deionized water and ethanol until the conductivity of its eluting solution reached below 100 μS/cm, and then dried at 80 °C for 24 h. Subsequently, 5 g of the dried algal sludge and cobalt nitrate hexahydrate were thoroughly ground. The mass of cobalt nitrate hexahydrate (the unit is g) was denoted as “x”, with the values being 0 (as a control), 1.0, 1.2, or 1.4. After that, the mixture was placed in a high-temperature tubular furnace for calcination at 950 °C under an argon atmosphere, with a heating rate of 5 °C/min, for a duration of 3 h. The material was then allowed to cool naturally to obtain x Co/SSPC (where x = 0, 1.0, 1.2, 1.4, representing the mass of cobalt nitrate hexahydrate, the corresponding mass fractions of Co are 0, 5.43%, 6.66%, and 7.63%, as shown in Supplementary Table S1).

### 2.3. Characterizations

Scanning electron microscopy (SEM) is a microscopic analysis technique used to analyze the microscopic morphology of materials. Transmission electron microscopy (TEM) is a microscopic technique that uses a high-energy electron beam to penetrate an ultra-thin sample for imaging, and it can provide high-resolution information about the internal structure of the material. Microscopic morphology images of samples were acquired using a SU8010 scanning electron microscope (SEM, SU8000, Hitachi Ltd., Tokyo, Japan) and a transmission electron microscope (TEM, Ecnai F20, JEOL Ltd., Tokyo, Japan). Raman spectra were obtained by means of a laser confocal Raman spectrometer equipped with a 532 nm laser (Raman, LabRAM Odyssey, Horiba, Loos, France). Selected area electron diffraction involves positioning the sample in a transmission electron microscope (TEM), determining the selected area, and then allowing the electrons within the selected area to pass through the aperture to participate in the diffraction process, thereby obtaining the electron diffraction pattern. Generally, it can be applied to the analysis of crystal structures, the identification of phases, the analysis of crystal orientations, as well as the study of interfaces and phase boundaries. X-ray diffraction (XRD) is a technique used to characterize the crystal structure of materials. X-ray diffraction patterns were acquired through the use of a D8-Focus X-ray diffractometer (XRD, D8 ADVANCE, Bruker AXS GmbH, Karlsruhe, Germany). For XRD, a Cu Kα radiation source (wavelength λ = 1.5406 Å) was used with a scanning range of 2θ = 10–80°, a scanning rate of 5°/min, a tube voltage of 40 kV, and a tube current of 40 mA. X-ray photoelectron spectroscopy (XPS) is a technique that can analyze the elemental composition and chemical state of the material surface. For XPS, an Al Kα radiation source (energy 1486.6 eV) was used with charge correction referenced to the C 1s peak (284.8 eV) as an internal standard. The analysis chamber was maintained under a vacuum better than 1 × 10−^9^ mbar. X-ray photoelectron spectroscopy (XPS) analysis of Co/SSPC was carried out on an X-ray photoelectron spectrometer (XPS, Kalpha, Thermo, Waltham, MA, USA). The XPS spectral data were processed using the Avantage 6.9 software. The baseline correction is carried out in the Shirley mode. During the peak fitting process, according to the symmetry and full width at half maximum characteristics of the characteristic peaks of different elements, the ratio of the Gaussian–Lorentz mixed peaks was optimized and adjusted within the range of 3:7 to 7:3. Meanwhile, the parameters such as peak area and binding energy were iteratively fitted by the least squares method. When the sum of squared residuals of the fitting (*R*^2^) is greater than 0.98 and the peak shape highly matches the original data, the fitting result is considered valid.

### 2.4. Measurements of Electromagnetic Parameters

Paraffin wax serves as a matrix material, which undertakes the task of supporting the samples and plays a crucial role in the sample preparation process for microwave absorption tests. The samples were uniformly mixed with paraffin wax at a mass ratio of 1:1 and subsequently pressed into annular samples with an inner diameter of 3.04 mm and an outer diameter of 7 mm. The coaxial method was adopted, and a vector network analyzer (Agilent N5234A, Agilent Technologies, Inc., Santa Clara, CA, USA) was utilized to measure the complex permittivity and the relative complex magnetic permeability of each sample of Co/SSPC within the frequency range of 2–18 GHz. The software will perform calculations and directly record the results of S-parameters, permittivity, and permeability. Based on the transmission line theory, the reflection loss (*RL*) values were calculated using the relevant formulas [33,34,35,36].

(1) $$RL\;\left(dB\right)=20log\left|\frac{Z_{in}-Z_{0}}{Z_{in}+Z_{0}}\right|\;,$$

(2) $$Z_{in}=Z_{0}\sqrt{\frac{\mu_{r}}{\varepsilon_{r}}}\tanh\left(j\frac{2\pi fd}{c}\sqrt{\frac{\mu_{r}}{\varepsilon_{r}}}\right)\;,$$

(3) $$\varepsilon_{r}=\varepsilon^{\prime }-j\varepsilon^{\prime \prime }\;,$$

(4) $$\mu_{r}=\mu^{\prime }-j\mu^{\prime \prime }\;,$$

where $Z_{in}$ is the input impedance and $Z_{0}$ is the air impedance, and are the relative permeability and permittivity, f is the frequency of the incident microwave, *c* is the speed of light in vacuum (3.0 × 10^8^ m/s), and *d* is the thickness of the microwave absorption material.

## 3. Results and Discussion

**Figure 1:**
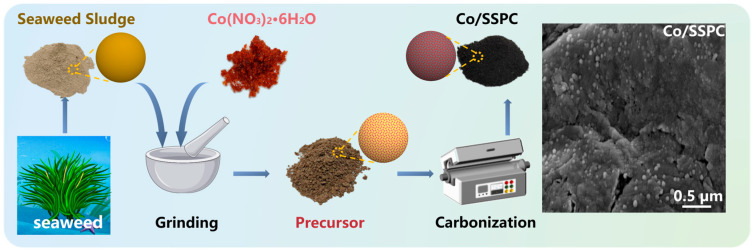
Schematic illustration of the preparation of Co-doped porous carbon derived from sea-weed sludge.

Algae sludge inherently contains self-doped substances and features a pore structure. The introduction of Co can synergize with carbon, compensating for the magnetic loss capability that carbon-based materials lack, which is conducive to enhancing the microwave-absorbing properties of the materials. Figure 1 depicts the preparation process of Co-doped porous carbon derived from algae sludge (Co/SSPC). Initially, the washed and dried seaweed sludge was ground together with cobalt nitrate hexahydrate. Subsequently, the mixture was placed in a tube furnace and calcined at 950 °C under an Ar atmosphere for 3 h to obtain Co-doped porous carbon with varying Co contents. In this process, during the carbonization of biomass polymers (such as cellulose and hemicellulose), decomposition will occur, and a part of the cobalt nitrate will also undergo thermal decomposition to form cobalt oxide. Then, cobalt oxide is reduced to elemental cobalt by carbon, with the release of carbon dioxide. During the pyrolysis of cellulose, it first undergoes depolymerization to produce low polymers such as glucose monomers, and then dehydration reactions occur to generate hydrogen. This hydrogen also reduces cobalt oxide to Co, accompanied by the formation of water vapor [31].

**Figure 2:**
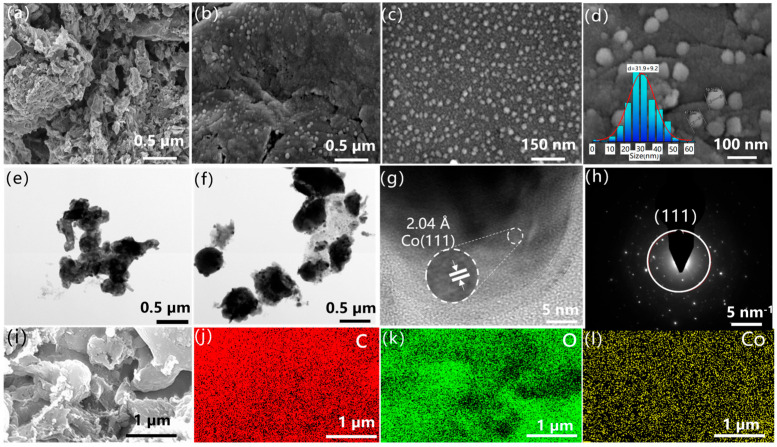
SEM images of (**a**) 0 Co/SSPC and (**b**–**d**) 1.0 Co/SSPC; TEM images of (**e**) 0 Co/SSPC and (**f**,**g**) 1.0 Co/SSPC; (**h**) SAED image of 1.0 Co/SSPC; (**i**–**l**) EDS mappings of 1.0 Co/SSPC.

To gain in-depth insights into the internal structure and micro-morphology of Co/SSPC, scanning electron microscopy (SEM) and transmission electron microscopy (TEM) characterizations were conducted. As shown in Figure 2, the porous carbon exhibits a disordered pore structure internally. When electromagnetic waves enter the material, the presence of pore channels causes them to undergo multiple reflections within the structure, a feature that enhances the multiple reflection loss of electromagnetic waves inside the material. Figure 2b–d illustrate that some granular nanoparticles are relatively uniformly distributed on the surface of the porous carbon, with their particle size distribution following a normal distribution and an average particle size of 31.9 nm (Figure 2d). As shown in Supplementary Figure S1, with the variation of Co concentration, the average particle size of the dominant population progressively increases from 31.9 nm to 36.7 nm and further to 48.7 nm. The increase in particle size gives rise to a decrease in interfacial density, thereby leading to a reduction in charge accumulation at the interface. This phenomenon is also reflected as a decline in the dielectric constant (εᵣ) and polarization loss, which may consequently cause a deterioration in microwave absorption performance [37]. The phase interfaces between the particles and the carbon skeleton are conducive to polarization effects. In Figure 2e,f, the dark regions are primarily composed of cobalt and inorganic salts, while the lighter areas are likely the carbon skeleton [38]. Figure 2g indicates that the Co/SSPC displays an irregular cladding structure. Its outer layer is composed of crystalline carbon, and within it, the (111) crystal plane of a cobalt single crystal is found [25].

From the selected diffractogram in Figure 2h, the Co/SSPC exists in a polycrystalline form internally. There are expected to be numerous phase interfaces between the crystals. Due to the differences in electrical conductivity and permittivity among Co crystals, carbon, and inorganic salts, charge accumulation occurs at their interfaces. This charge accumulation results in the distortion of the electric field, thereby causing interfacial polarization [39].

**Figure 3:**
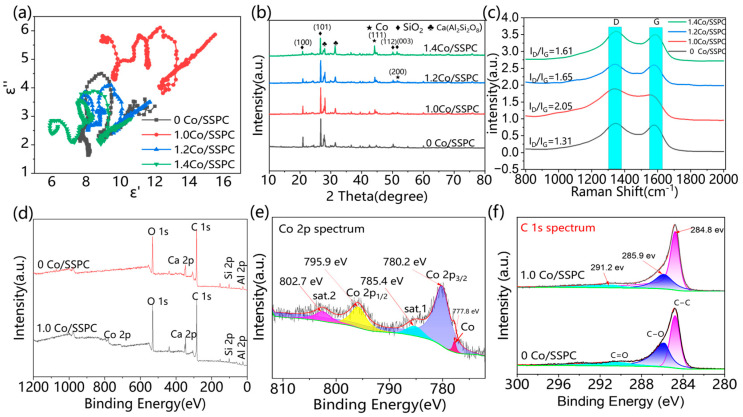
(**a**) Cole-Cole diagram of the Co/SSPC composite; (**b**) XRD and (**c**) Raman spectra of the Co/SSPC composite; (**d**) XPS survey spectrum of 0 Co/SSPC and 1.0 Co/SSPC; (**e**,**f**) high-resolution spectra of (**e**) Co 2p and (**f**) C 1s.

To understand the crystalline components of Co/SSPC, the crystal composition of the material was determined via X-ray diffraction (XRD, Figure 3b). To further investigate the elemental composition and chemical valence states of Co/SSPC, X-ray photoelectron spectroscopy (XPS, Figure 3d) was used for characterization. The main phases in the Co-doped porous carbon derived from algal sludge are SiO_2_, Ca(Al_2_Si_2_O_8_), and Co. This indicates the existence of inherent doping substances within the algal biomass, which contribute to polarization loss. The diffraction peaks at 44.1° and 51.4° (Supplementary Table S2) correspond to the (111) and (200) crystal planes of Co, respectively, confirming the presence of metallic cobalt crystals. These peaks are generally consistent with the values of 44.2° and 51.5° in the standard card JCPDS No. 15-0806 for Co. The minor discrepancy is likely due to instrument errors. As the intensity of the characteristic peak of Co increases with the doping amount, it indicates an improvement in the crystallinity of Co, which is consistent with the slight enhancement trend of magnetic loss in microwave absorption performance.

Raman spectroscopy provides information about the molecular structure, chemical bonds, and crystal lattice of materials. Through Raman spectroscopy, we can obtain the proportion of graphitized and non-graphitized components in the carbon material sample, and then calculate the graphitization degree of the sample. In the Raman spectra (Figure 3c), a characteristic D band at approximately 1350 cm^−1^ and a G band at around 1590 cm^−1^ signify the graphitized structure of the carbon matrix [40]. The intensity ratio (I_D_/I_G_) offers insights into the degree of disorder or graphitization of the carbon structure. In microwave-absorbing materials, to achieve good microwave absorption performance, an appropriate ratio of amorphous carbon to crystalline car-bon is required to obtain suitable dielectric constants. If the material is entirely composed of crystalline carbon, it exhibits good electrical conductivity. Due to the skin effect, electromagnetic waves will be completely reflected. On the contrary, if it is entirely amorphous carbon with poor electrical conductivity, the skin effect does not occur at all, and electromagnetic waves will completely penetrate the material. As the Co content increases from 1.0 to 1.4, the I_D_/I_G_ ratio decreases from 2.05 to 1.61, suggesting enhanced graphitization of Co-SSPC and a reduction in amorphous carbon content. This can be attributed to the fact that the introduction of cobalt induces the material to preferentially form a coke-like structure. With an increase in cobalt content, the amount of crystalline carbon increases, thereby enhancing the degree of graphitization [41]. Graphite crystals feature a typical layered structure, which allows electrons to move relatively freely between the layers. As the degree of graphitization increases, the electrical conductivity of the Co/SSPC also increases. When electromagnetic waves irradiate the surface, the conduction current generates a magnetic field opposing the incident electromagnetic wave. Simultaneously, the degree of graphitization exerts an impact on the dielectric constant and dielectric loss of C_O_/SSPC. Under the influence of electromagnetic waves, dipoles and charged particles within the C_O_/SSPC are more prone to experiencing polarization and relaxation phenomena. During this process, a fraction of the electromagnetic energy is converted into thermal energy and dissipated [42,43].

To achieve enhanced microwave-absorbing performance, proper impedance matching and dielectric loss must be meticulously coordinated. When comparing the Co/SSPC with the Co-free SSPC, it is expected that the impedance matching and dielectric loss of the Co/SSPC are better coordinated, thereby leading to an improvement in its wave-absorbing performance. Nevertheless, as the Co content increases, the degree of graphitization increases. This increase may give rise to an increase in the conduction current on the material surface, consequently generating a stronger reverse magnetic field. Such a stronger reverse magnetic field will undermine the coordination between impedance matching and dielectric loss, ultimately resulting in a deterioration of the wave-absorbing performance.

Figure 3d presents the full-scan energy spectrum of Co/SSPC, revealing the elemental composition of the material. The detected elements are C, O, Ca, Si, Al, and Co, respectively. Subsequently, the high-resolution spectra of C 1s and Co 2p were fitted using a fitting method. In Figure 3e, the characteristic peaks correspond to 2p1/2 and 2p3/2 of the Co element, with peak values of 780.2 eV and 795.9 eV, respectively. This indicates that Co mainly exists in the form of Co^2^⁺, and there is also the presence of a Co singlet, which is consistent with the previous XRD characterization results. The percentages of the Co 2p_3_/_2_ peak, sat.1 peak, Co 2p_1_/_2_ peak, sat.2 peak, and Co peak are 55%, 12%, 20%, 8%, and 5%, respectively (Supplementary Table S3). In Figure 3f, the characteristic peaks of C 1s correspond to C-C, C-O, and C=O, respectively. The peak area percentages of C-C, C-O and C=O for 0 Co/SSPC are 65%, 25% and 10%, respectively; those for 1.0 Co/SSPC are 77%, 14% and 9%, respectively. After the addition of cobalt, the peak area of C-O decreased from 25% to 14% (Supplementary Table S4). This decrease may be attributed to the consumption of some carbon by CoO, which corroborates the previous findings. The presence of C-O and C=O functional groups in the C1s spectrum indicates that the biomass polymer retains partial polar groups during the carbonization process, which helps enhance the dipole polarization loss capability of the material.

**Figure 4:**
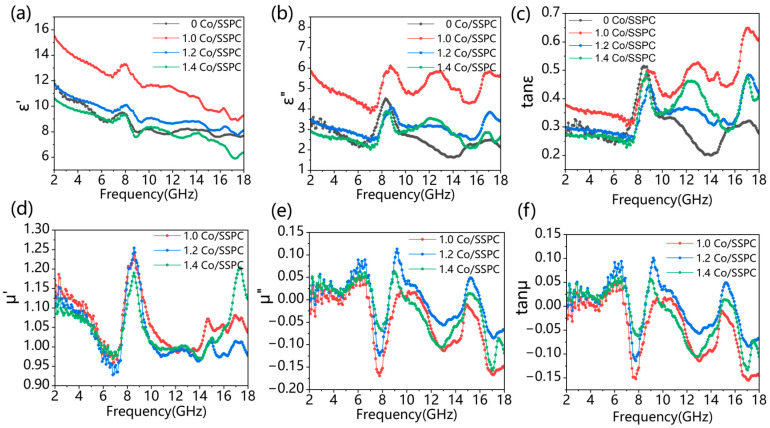
(**a**,**b**) Complex permittivity, (**d**,**e**) complex permeability, (**c**) dielectric loss, and (**f**) magnetic loss of Co/SSPC composite.

As depicted in Figure 4, the magnetic permeability curves of 1.0 Co/SSPC, 1.2 Co/SSPC, and 1.4 Co/SSPC are intertwined, suggesting comparable magnetic losses among these samples. The primary factor contributing to the variation in microwave-absorption performance is likely the dielectric constant. As shown in Figure 4a, the real part of the dielectric constant decreases with an increase in frequency. This decrease may be attributed to the fact that the introduction of Co has altered the impedance matching of the material. In Figure 4b, the imaginary part of the dielectric constant experiences a sudden increase around 8, 13, and 17 GHz. This phenomenon could be due to the material’s polarization mechanism being unable to keep pace with the rapid changes in the electric field. Consequently, this also leads to a sudden increase in the curve in Figure 4c at 8, 13, and 17 GHz. The magnetic loss of Co-free SSPC is zero. However, the addition of cobalt imparts a certain degree of magnetic loss to the Co doped SSPC. According to Debye relaxation theory, the relationship between $\varepsilon^{\prime }$ and $\varepsilon^{\prime \prime }$ can be explained by [36,43,44]:

(5) $${\left(\varepsilon^{\prime }-\frac{\varepsilon_{s}+\varepsilon_{\infty }}{2}\right)}^{2}+{\left(\varepsilon^{\prime \prime }\right)}^{2}={\left(\frac{\varepsilon_{s}-\varepsilon_{\infty }}{2}\right)}^{2},$$

where $\varepsilon_{s}$ represents the static dielectric constant, while $\varepsilon_{\infty }$ denotes the optical dielectric constant. The Cole–Cole semicircle is employed to characterize the polarization relaxation process, where each semicircle represents a Debye relaxation process. Meanwhile, the linear relationship between the real part and the imaginary part of the complex permittivity indicates the presence of conductive loss. Figure 3a showcases the Cole–Cole curves of Co/SSPC composites with varying Co contents. These curves are complex and distorted, signifying the co-existence of multiple loss mechanisms, such as dipole polarization and interfacial polarization. In the high-frequency region, the linear segment validates the existence of conductive loss. As the Co content increases, the length of this linear segment diminishes, corresponding to a reduction in conductive loss. This reduction in conductive loss may be attributed to the change in the ratio of graphitized carbon to amorphous carbon, as previously shown in Figure 3c [45].

**Figure 5:**
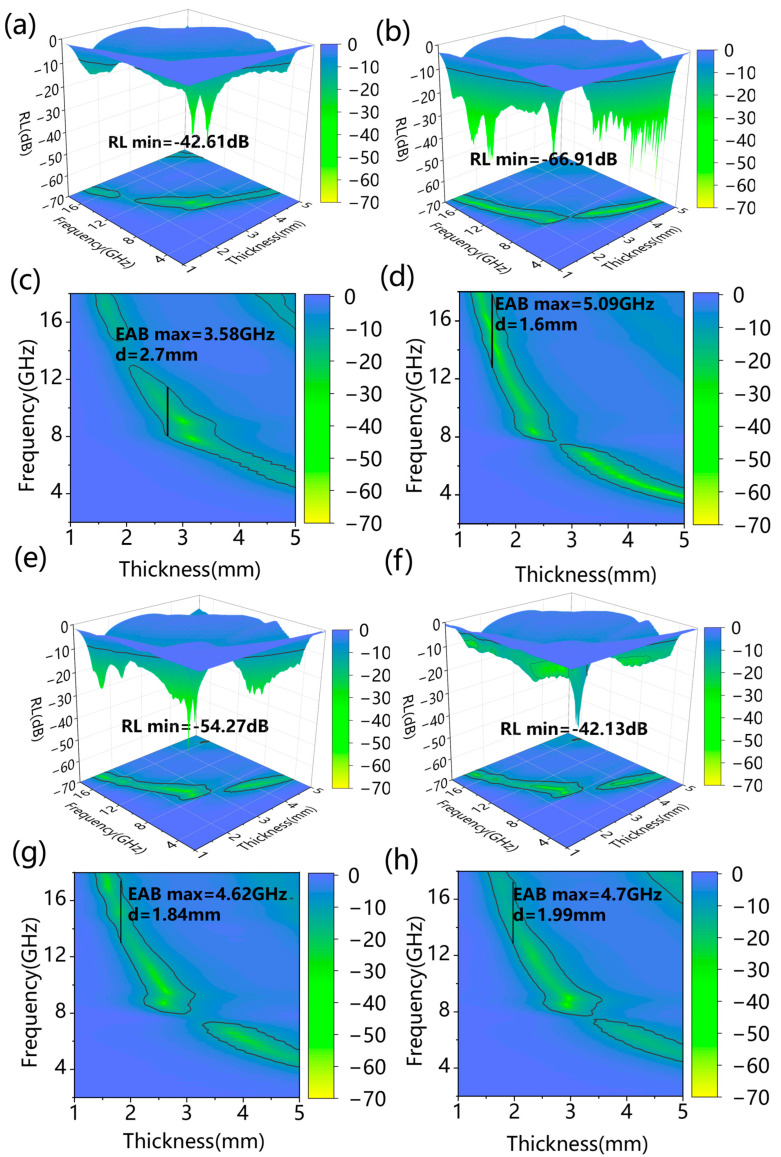
The reflection loss 3D map of (**a**) 0 Co/SSPC, (**b**) 1.0 Co/SSPC, (**e**) 1.2 Co/SSPC, and (**f**) 1.4 Co/SSPC; the effective absorption band 2D map of (**c**) 0 Co/SSPC, (**d**) 1.0 Co/SSPC, (**g**) 1.2 Co/SSPC, and (**h**) 1.4 Co/SSPC.

**Figure 6:**
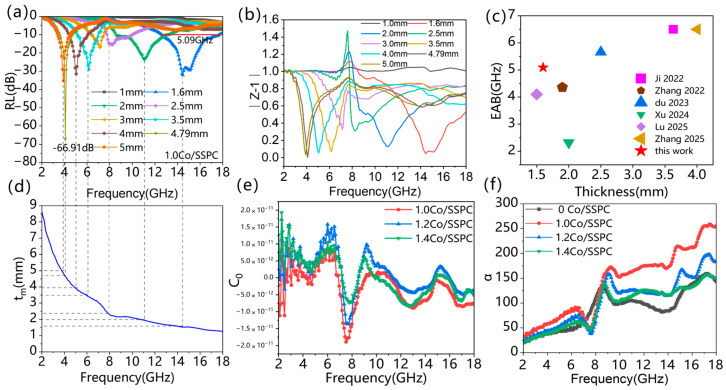
(**a**) The reflection loss curves of 1.0 Co/SSPC, (**b**) |*Z* − 1| represents the impedance matching curve of 1.0 Co/SSPC, (**c**) The EAB comparison between previous works and this work: Ji 2022 [46], Zhang 2022 [47], Du 2023 [48], Xu 2024 [49], Lu 2025 [51], Zhang 2025 [52], (**d**) the corresponding calculated matching thickness tm for RL peaks of 1.0 Co/SSPC, (**e**) *C*_0_ represents the eddy current loss values of 1.0 Co/SSPC, 1.2 Co/SSPC, and 1.4 Co/SSPC, (**f**) α represents the attenuation coefficient of 1.0 Co/SSPC.

Figure 5 presents the 3D reflection loss plot and 2D absorption bandwidth plot of the Co/SSPC composite. It is revealed that 0 Co/SSPC attained a maximum effective absorption bandwidth (EAB_max_) of 3.58 GHz at a thickness of 2.7 mm, with a minimum reflection loss (RL_min_) of −42.61 dB. As anticipated, the 1.0 Co/SSPC achieved a higher EAB_max_ of 5.09 GHz at a thickness of 1.6 mm, accompanied by a lower RL_min_ of −66.91 dB, while the 1.2 Co/SSPC and 1.4 Co/SSPC reached a EAB_max_ of approximately 4.62 GHz, with an RL_min_ of −54.27 dB and −42.13 dB, respectively. Overall, as the Co content increases, the microwave absorption performance initially increases and then decreases, which is consistent with the variation in the ratio of amorphous-to-crystalline carbon, as previously discussed in Figure 3c. Furthermore, it is observable that the Co/SSPC displays almost no wave-absorbing capacity in the vicinity of 8 GHz. This phenomenon can be ascribed to the poor impedance matching of the material at this specific frequency. As shown in Figure 6c, when comparing our work with similar previously published works, taking into account the microwave-absorbing performance, our ultra-thin matching thickness is particularly prominent. This indicates that the prepared Co/SSPC has the potential to be employed as a microwave absorber. As shown in Table 1, compared with some recently published microwave-absorbing materials based on biomass-derived carbon, the Co/SSPC composite exhibits superior performance in terms of effective absorption bandwidth, reflection loss value, and matching thickness.

**Table 1:** Performance of recently reported microwave-absorbing materials based on biomass-derived carbon.

Materials	RL_min_ (dB)	Thickness (mm)	EAB_max_ (GHz)	Thickness (mm)	Ref.
BC/CoFe	−54.4	2.2	2.6	2.4	[46]
Hierarchically porous PNC	−56.3	1.4	3.44	1.4	[47]
Fe_3_C/Fe@NBPC	−52.25	2.71	3.06	2.71	[48]
Biomass derived PANI/BPC	−40.89	2.6	4.24	2.1	[49]
MoS_2_/CCFs	−39.1	1.7	4.4	1.7	[50]
C/CoNi	−54.59	-	3.96	-	[51]
Co/SSPC	−66.91	4.79	5.09	1.6	This work

Figure 6a,d show the 2D wave absorption line graphs and 1/4 wavelength theoretical curves of Co/SSPC composite. As the matching thickness is incrementally increased, the absorption performance gradually shifts towards the lower-frequency band (Figure 6a). By adjusting the matching-thickness value, it is possible to achieve full-absorption loss within the 2–18 GHz frequency range. Furthermore, the reflection-loss values corresponding to different matching thicknesses can be correlated with the 1/4 λ curves (Figure 6d). This correlation indicates that the Co/SSPC composites are in accordance with the 1/4-wavelength theoretical model. According to the principle of wave interference, when the phase difference between two waves is such that it is equivalent to a path-difference of half a wavelength, destructive interference occurs. In a microwave-absorbing material with a thickness equal to a quarter-wavelength, the phase difference between the reflected waves at the air/absorber interface and the absorber/metal-backplane interface satisfies the condition for destructive interference. The phase difference resulting from the path difference leads to their mutual cancellation. The model is as follows [52]:

(6) $$t_{m}=\frac{nc}{4f\sqrt{\left|\mu_{r}\right|\left|\varepsilon_{r}\right|}}\left(n=1,\;3,\;5\ldots \ldots \right),$$

where *t_m_* denotes the matched thickness of RL_min_. A good microwave-absorbing material should have an optimized impedance matching to achieve more electromagnetic waves into the interior of the material. The impedance matching equation is as follows [52]:

(7) $$Z=\frac{Z_{in}}{Z_{0}}\;,$$

(8) $$\left|Z-1\right|=\left|\frac{Z_{in}}{Z_{0}}-1\right|\;,$$

where *Z_in_* denotes the input impedance, *Z*_0_ denotes the free-space impedance. From Figure 6b, the material presents better impedance matching performance in general, and its *Z* − 1 value is closest to 1 at matching thicknesses of 1.6 mm, 4.79 mm, and 5.0 mm, showing better electromagnetic wave absorption performance. When the alternating magnetic field is perpendicular to the metal surface, an induced electromotive force will be generated inside the metal block. Since the metal is a conductor, this induced electromotive force will drive free electrons to form a circular current, that is, an eddy current [53]. The eddy current loss value $C_{0}\;$ of the material was calculated according to the following equation [37,54,55]:

(9) $$C_{0}=\frac{\mu^{\prime \prime }}{{\left(\mu^{\prime }\right)}^{2}f}\;,$$

According to the analytical equation, if the magnetic losses are all from eddy current losses, then the $C_{0}\;$ value should remain constant with frequency. By observing the $C_{0}$ curve in Figure 6e, it remains constant at around 0. It shows that the magnetic loss of the material is mainly eddy current loss, and there is a weak magnetic resonance loss. The attenuation coefficient is one of the criteria for evaluating the electromagnetic wave absorption performance of microwave-absorbing materials, which is calculated as follows according to the following equation [40,56]

(10) $$\alpha =\frac{\sqrt{2}\pi f}{c}\sqrt{(\mu^{\prime \prime }\varepsilon^{\prime \prime }-\mu^{\prime })+\sqrt{{\left(\mu^{\prime \prime }\varepsilon^{\prime \prime }-\mu^{\prime }\varepsilon^{\prime }\right)}^{2}+{\left(\mu^{\prime }\varepsilon^{\prime \prime }-\mu^{\prime \prime }\varepsilon^{\prime }\right)}^{2}}}\;,$$

where $\varepsilon^{\prime },\varepsilon^{\prime \prime },\mu^{\prime }$ and $\mu^{\prime \prime }\;$ are electromagnetic parameters, *f* is the frequency and *c* is the speed of light. The larger the value of the attenuation coefficient, the stronger the ability of the composite material to attenuate electromagnetic waves [57]. As can be observed from Figure 6f, the attenuation coefficients of different Co/SSPC composites initially increase and then decrease as the frequency ascends. Among them, the attenuation coefficient of 1.0 Co/SSPC is the largest. This indicates that the 1.0 Co/SSPC material exhibits a relatively high level of internal loss. Consequently, a substantial amount of the electromagnetic wave’s energy can be absorbed and dissipated after the wave travels a short distance within the material. This phenomenon also demonstrates that the coordination between impedance matching and internal loss within the 1.0 Co/SSPC material is relatively favorable, which corroborates the previous assertions.

**Figure 7:**
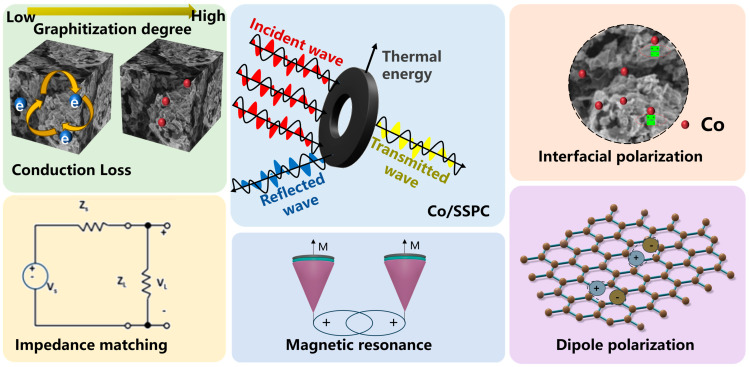
Schematic illustration of the microwave absorption mechanisms of Co/SSPC.

Furthermore, based on the above-mentioned experimental results, the possible microwave-absorbing mechanism of Co/SSPC was proposed, as shown in Figure 7. Firstly, the impedance characteristics of Co/SSPC are determined by its composition and microstructures. When an incident electromagnetic wave impinges upon the material surface, only a fraction of the electromagnetic wave is reflected, while another portion of the wave can penetrate the material unhindered for absorption [58]. Within the material, there exist numerous micro-scale inhomogeneous structures. These structures cause the electromagnetic wave to undergo multiple reflections and attenuation processes within the material [59].

Secondly, the Co/SSPC encompasses a variety of substances with distinct properties, including self-doped metal oxides, SiO_2_, and Co. These substances exhibit different crystal structures, electron-cloud distributions, and atomic arrangements, thereby giving rise to varying electromagnetic properties, such as differences in dielectric constant and electrical conductivity. Upon exposure to an electromagnetic wave, the charges at the interfaces of these substances are redistributed, leading to the formation of electric dipoles. These electric dipoles continuously realign their orientations in response to the changing electric field. During this process, electromagnetic energy is dissipated, resulting in interfacial polarization loss [60].

Thirdly, oxygen atoms possess a high degree of electronegativity. Within the atomic structure of the Co/SSPC, they attract the surrounding electron clouds, leading to an uneven charge distribution in their vicinity. When subjected to an electromagnetic wave, the rotation of oxygen-related electric dipoles demands energy consumption, thereby giving rise to dipole polarization [60].

Finally, metallic Co is uniformly dispersed throughout the material and interweave with the carbon skeleton, thereby constructing an efficient conductive network for the Co/SSPC. When an electromagnetic wave penetrates the material, the electric field it conveys propels the free electrons within the conductive network to move directionally. This movement forms an electric current, which subsequently generates Joule heat, giving rise to conduction loss [61]. Moreover, the Co element also endows the Co/SSPC composite with eddy-current loss and weak magnetic resonance loss [62].

In general, the outstanding microwave-absorption performance of Co/SSPC can be ascribed to the combined effect of multiple absorption mechanisms. These mechanisms achieve a synergistic enhancement of magnetic loss and dielectric loss, leading to the remarkable performance of the material in microwave absorption.

## 4. Conclusions

In summary, it was found that Co can simultaneously regulate the dielectric and magnetic properties of the composite. Meanwhile, using biomass polymers as precursors offers advantages in terms of cost and sustainable development. It was successfully synthesized cobalt-doped seaweed sludge porous carbon (Co/SSPC) with different cobalt contents through a simple grinding-carbonization process. The introduction of cobalt enhances dielectric loss and impedance matching by adjusting the ratio of crystalline to amorphous carbon, while endowing the porous carbon with magnetic loss capabilities. This establishes a synergistic mechanism of dielectric/magnetic loss, thereby improving its microwave absorption performance. The results show that the 1.0 Co/SSPC composite exhibits excellent absorption performance, with the minimum reflection loss of −66.91 dB at a thickness of 4.79 mm and an effective absorption bandwidth of 5.09 GHz at a thickness of 1.6 mm. The synergistic effect between the magnetic loss caused by cobalt and the dielectric loss of porous carbon is crucial for improving the microwave absorption performance. This study not only demonstrates a feasible approach for the functional utilization of waste algal sludge but also highlights its potential in the low-cost production of microwave-absorbing materials.

## Data Availability

Data are contained within the article.

## Supplementary Materials

The following supporting information can be downloaded at: https://www.mdpi.com/article/10.3390/polym17111572/s1, Figure S1: SEM images of (a) 1.2 Co/SSPC and (b) 1.4 Co/SSPC; Table S1: Element concentrations of some elements in Co/SSPC; Table S2: The values of the peak centers of different crystal planes of Co and SiO_2_; Table S3: The percentage of different sub-peaks of Co; Table S4: The percentage of different chemical bonds in C1s.
