# Supplementary material for: Graphitization Optimization of Cobalt-Doped Porous Carbon Derived from Seaweed Sludge for Enhanced Microwave Absorption
Source: Polymers (Basel). 2025 Jun 5;17(11):1572. doi: 10.3390/polym17111572 (PMC12157164; doi:10.3390/polym17111572)
Supplement: Supplementary file 1 [file polymers-17-01572-s001.zip › polymers-3623440-supplementary.pdf]

Supporting information

Graphitization Optimization of Cobalt - Doped Porous Carbon Derived from Seaweed Sludge for Enhanced Microwave Absorption

Kai Liu [1], Yusen Ai [2], Mei Cui [2], Renliang Huang [1,*] and Rongxin Su [1,2,3,*]

[1] Tianjin Key Laboratory for Marine Environmental Research and Service, School of Marine Science and Technology, Tianjin University, Tianjin, 300072, PR China; lk66_99@tju.edu.cn
[2] State Key Laboratory of Chemical Engineering, Tianjin Key Laboratory of Membrane Science and Desalination Technology, School of Chemical Engineering and Technology, Tianjin University, Tianjin, 300072, PR China
[3] Zhejiang Institute of Tianjin University, Ningbo, Zhejiang, 315201, China
* Correspondence: tjuhrl@tju.edu.cn ; surx@tju.edu.cn

Table S1. Element concentrations of some elements in Co/SSPC.

Element	Co	Si	Al	Ca
0 Co/SSPC	0	12%	3.67%	12.7%
1.0 Co/SSPC	5.43%	14.4%	4.21%	11.2%
1.2 Co/SSPC	6.66%	13.4%	3.97%	10.8%
1.4 Co/SSPC	7.63%	9.16%	3.73%	9.26%

Table S2. The values of the peak centers of different crystal planes of Co and SiO_2.

	Co(111)	Co(200)	SiO_2(100)	SiO_2(101)	SiO_2(112)	SiO_2(003)
Peak center intensity	44.1°	51.4°	20.9°	26.7°	50.1°	51.4°

Table S3. The percentage of different sub - peaks of Co

	Co 2p1/2	Sat.1	Co 2p1/2	Sat.2	Co
percentage	55%	12%	20%	8%	5%

Table S4. The percentage of different chemical bonds in C1s

Sample	C - C	C - O	C = O
1.0 Co/SSPC	77%	14%	9%
0 Co/SSPC	65%	25%	10%

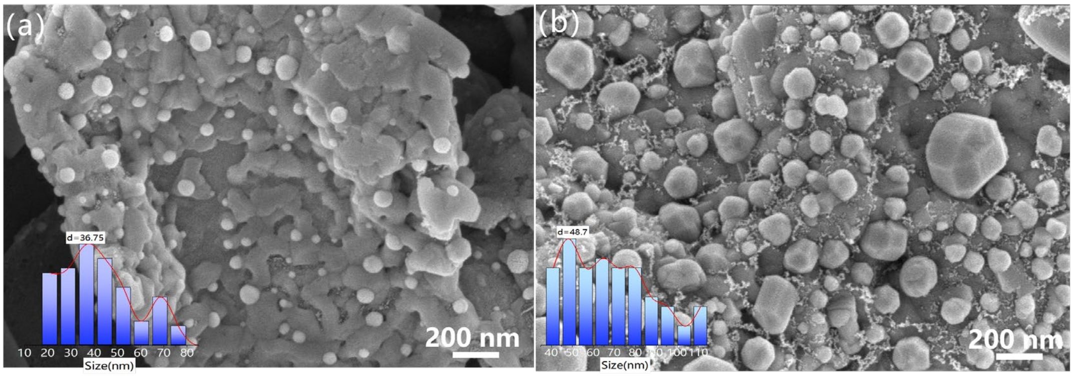

Figure S1. SEM images of (a) 1.2 Co/SSPC and (b) 1.4 Co/SSPC
